# Large Language Models for the National Radiological Technologist Licensure Examination in Japan: Cross-Sectional Comparative Benchmarking and Evaluation of Model-Generated Items Study

**DOI:** 10.2196/81807

**Published:** 2025-11-13

**Authors:** Toshimune Ito, Toru Ishibashi, Tatsuya Hayashi, Shinya Kojima, Kazumi Sogabe

**Affiliations:** 1 Department of Radiological Technology, Faculty of Medical Technology, Teikyo University, 2-11-1 Kaga, Itabashi-ku, Tokyo, 173-8605, Japan, +81-3-3964-7053; 2 Department of Medical Radiology, Graduate School of Medical Technology, Teikyo University, Tokyo, Japan; 3 Department of Medical Radiological Technology, Faculty of Health Sciences, Kyorin University, Tokyo, Japan; 4 Department of Radiology, Tokyo Women’s Medical University Adachi Medical Center, Tokyo, Japan; 5 Department of Radiological Sciences, School of Health Sciences, Ibaraki Prefectural University of Health Sciences, Ibaraki, Japan

**Keywords:** large language models, licensing exam, radiology, educational evaluation, medical education, item generation

## Abstract

**Background:**

Mock examinations are widely used in health professional education to assess learning and prepare candidates for national licensure. However, instructor-written multiple-choice items can vary in difficulty, coverage, and clarity. Recently, large language models (LLMs) have achieved high accuracy in medical examinations, highlighting their potential for assisting item-bank development; however, their educational quality remains insufficiently characterized.

**Objective:**

This study aimed to (1) identify the most accurate LLM for the Japanese National Examination for Radiological Technologists and (2) use the top model to generate blueprint-aligned multiple-choice questions and evaluate their educational quality.

**Methods:**

Four LLMs—OpenAI o3, o4-mini, o4-mini-high (OpenAI), and Gemini 2.5 Flash (Google)—were evaluated on all 200 items of the 77th Japanese National Examination for Radiological Technologists in 2025. Accuracy was analyzed for overall items and for 173 nonimage items. The best-performing model (o3) then generated 192 original items across 14 subjects by matching the official blueprint (image-based items were excluded). Subject-matter experts (≥5 y as coordinators and routine mock examination authors) independently rated each generated item on five criteria using a 5-point scale (1=unacceptable, 5=adoptable): item difficulty, factual accuracy, accuracy of content coverage, appropriateness of wording, and instructional usefulness. Cochran Q with Bonferroni-adjusted McNemar tests compared model accuracies, and one-sided Wilcoxon signed-rank tests assessed whether the median ratings exceeded 4.

**Results:**

OpenAI o3 achieved the highest accuracy overall (90.0%; 95% CI 85.1%‐93.4%) and on nonimage items (92.5%; 95% CI 87.6%‐95.6%), significantly outperforming o4-mini on the full set (*P*=.02). Across models, accuracy differences on the non-image subset were not significant (Cochran Q, *P*=.10). Using o3, the 192 generated items received high expert ratings for item difficulty (mean, 4.29; 95% CI 4.11‐4.46), factual accuracy (4.18; 95% CI 3.98‐4.38), and content coverage (4.73; 95% CI 4.60‐4.86). Ratings were comparatively lower for appropriateness of wording (3.92; 95% CI 3.73‐4.11) and instructional usefulness (3.60; 95% CI 3.41‐3.80). For these two criteria, the tests did not support a median rating >4 (one-sided Wilcoxon, *P*=.45 and *P*≥.99, respectively). Representative low-rated examples (ratings 1‐2) and the rationale for those scores—such as ambiguous phrasing or generic explanations without linkage to stem cues—are provided in the supplementary materials.

**Conclusions:**

OpenAI o3 can generate radiological licensure items that align with national standards in terms of difficulty, factual correctness, and blueprint coverage. However, wording clarity and the pedagogical specificity of explanations were weaker and did not meet an adoptable threshold without further editorial refinement. These findings support a practical workflow in which LLMs draft syllabus-aligned items at scale, while faculty perform targeted edits to ensure clarity and formative feedback. Future studies should evaluate image-inclusive generation, use Application Programming Interface (API)-pinned model snapshots to increase reproducibility, and develop guidance to improve explanation quality for learner remediation.

## Introduction

Mock examinations are a key pedagogical tool in training programs for health professionals. These are designed to consolidate the knowledge required for national licensure and to gauge students’ achievement [1-3]. In particular, multiple-choice formats are valuable because they enable the systematic, efficient appraisal of the broad foundational knowledge expected in clinical practice, making them integral to the quality of the curriculum. However, most items are written by individual instructors that draw on past examinations or personal clinical experience, and their difficulty and content validity are rarely subjected to systematic review [4,5]. These can result in biases in content coverage, inconsistencies in wording, and variable educational usefulness, which undermine the stability of learning outcome assessments.

Several studies have reported the high accuracy of large language models (LLMs) in health professional licensure examinations, owing to their rapid advancements [6-9]. In text-based multiple-choice questions, models have begun to match or surpass human test-takers while generating rationales and keyword-level explanations that can serve as formative feedback [10-13]. These suggest the potential utility of LLM-assisted item writing during the construction of high-quality question banks. However, most research has centered on the accuracy of LLMs in answering existing licensure items [14-16], while empirical evidence regarding the educational quality of questions authored by LLMs remains scarce [13,17]. A comprehensive appraisal that includes (1) appropriate difficulty, (2) completeness and accuracy of content coverage, (3) clarity of option wording, and (4) usefulness of accompanying explanations is necessary to address this knowledge gap and clarify the practical value of artificial intelligence (AI)-supported mock examinations, as well as its limitations.

This study evaluated the quality of AI-generated multiple-choice questions based on the Japanese National Examination for Radiological Technologists. Several LLMs were used to answer the exam, then the highest-performing model was used to generate a set of mock items. These AI-generated questions were then evaluated across several aspects (ie, item-level difficulty, item-level factual accuracy, accuracy of content coverage, appropriateness of wording, and instructional usefulness) through blinded expert review and statistical analysis. By doing so, this study aims to provide empirical data on the educational soundness of AI-generated items, as well as highlight any emerging challenges.

## Methods

### Models and Study Period

Four LLMs released in February 2025 were evaluated: OpenAI o3, OpenAI o4-mini, OpenAI o4-mini-high (all OpenAI), and Gemini 2.5 Flash (Google). The evaluations were conducted from March 14 to May 8, 2025, using the publicly accessible browser interfaces, with the desired engine explicitly selected in each platform’s menu. The browser access was chosen to mirror typical educational use and to simplify image I/O (upload, preview, and per-item attachment). The item-generation study was conducted from May 15 to June 28, 2025, using OpenAI o3, the model with the highest answer accuracy. To ensure consistency, we used an identical Japanese prompt template across models. To avoid carryover effects, we started a new session for each 50-item batch with the OpenAI models and used per-item input with Gemini; image files (PNG) were attached when required by an item. As browsing and memory features were disabled, outputs relied solely on pretrained parameters and the provided materials.

### Answer Accuracy

**Figure 1.**
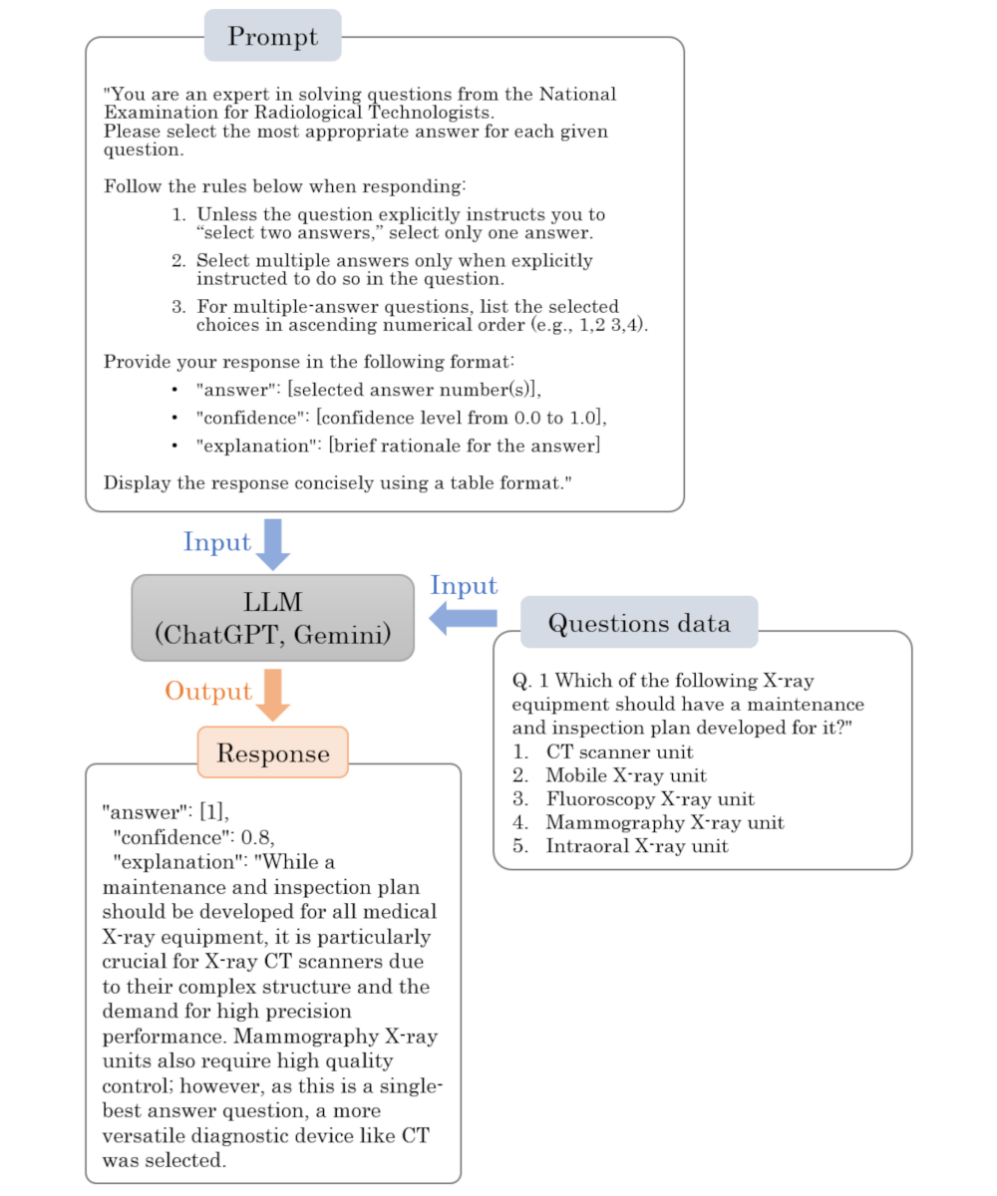
Representative interaction with a large language model (LLM). This diagram illustrates the workflow used to evaluate the answer accuracy of large language models. The LLMs were given prompts to answer each question (including text and images when applicable) in Japanese, with specific instructions for answer selection and formatting. The output included the selected answer, a confidence score, and a brief explanation. All actual prompts and inputs were entered in Japanese, but this example is shown in English for illustration purposes. CT: computed tomography.

Answer accuracy was assessed based on all 200 items of the 77th National Examination for Radiological Technologists, administered on February 20, 2025. All items were multiple-choice, and question stems containing images were presented unchanged. Each model was given the question stem and options in Japanese, then instructed to select the correct answers in single-best or multiple-select format. Multimedia Appendix 1 lists the subjects and the number of items per subject. Due to the differences in each model, the input procedures were adapted accordingly. For OpenAI models, stems and options were pasted from four text files (items 1‐50, 51‐100, 101‐150, and 151‐200) into separate sessions. PNG files were attached for each image item, with the filenames labeled to match the corresponding item numbers. However, since Gemini permits only one file upload, the stems and options were pasted directly into the prompt while attaching an image file as needed. All inputs were entered manually. A concrete workflow is shown in Figure 1.

The outputs of the model were compared to the official answer key issued by the Ministry of Health, Labor and Welfare. The correct and incorrect responses were counted overall for 200 items and separately for the 173 items that did not require image interpretation (ie, nonimage items). Statistical significance was tested across models.

### Item Generation

#### Generation Procedure

The mock items were generated using OpenAI o3, since it had the highest accuracy among all four models. Image-based stems were excluded since all models performed poorly on these. Using the same examination as a blueprint, OpenAI o3 was used to produce 192 questions across 14 subjects (Table 1), matching the same distribution of items. The model was supplied with text files containing the past 5 years of examination items and the official test specifications, ensuring its alignment with test objectives. Browsing remained disabled. Since Healthcare Safety Management is a new domain introduced in 2025, thereby lacking any historical reference items, it was excluded from the mock item generation. Items were generated separately for each subject in Japanese, and each output included the stem, five options, the key, and a brief rationale.

**Table 1.** Distribution of artificial intelligence (AI)-Generated Mock Items.

Subject	Blueprint target (n=200)	Generated (n=192)
Diagnostic Imaging Techniques	20	20
Nuclear Medicine Technology	20	20
Radiation Therapy Technology	20	20
Medical Imaging Informatics	10	10
Basic Medical Sciences	30	30
Radiation Science & Engineering	36	36
X-ray Imaging Equipment	20	20
X-ray Imaging Techniques	20	20
Image Engineering	6	6
Radiation Safety Management	10	10
Healthcare Safety Management^a^	8	0

a Since Healthcare Safety Management was only recently introduced as a new subject in the 2025 blueprint, it was excluded from the mock item generation.

### Evaluation of Generated Items

All 192 generated questions were reviewed by experts of the subject matter; these were faculty members with at least 5 years of experience as subject coordinators in radiological technology programs and who routinely author mock examinations. Items were assigned to reviewers by discipline, and each question was evaluated by one expert. The reviewers rated each item on a five-point scale: (1) unacceptable; (2) major revision needed; (3) revisable; (4) minor revision; and (5) adoptable across five criteria including, item difficulty, factual accuracy, accuracy of content coverage, appropriateness of wording, and instructional usefulness.

For each criterion, we calculated the median score and tested the statistical significance of the proportion of high ratings (≥4). The evaluation framework, which is based on faculty experience with national examination item writing, is presented in Table 2.

**Table 2.** Evaluation of generated items.

Evaluation criterion	Rating scale^a^
Item difficulty	1‐5
Factual accuracy	1‐5
Accuracy of content coverage	1‐5
Appropriateness of wording	1‐5
Instructional usefulness	1‐5

a Rating scale definition: 1=Unacceptable; 2=Major revision needed; 3=Revisable; 4=Minor revision; 5=Adoptable.

### Statistical Analysis

Statistical analysis was performed using JMP (version 18; JMP Statistical Discovery LLC). Cochran Q test was initially used to examine overall differences in answer accuracy; when significant, pairwise differences were probed with McNemar test using Bonferroni correction. The item generation study used a one-sided Wilcoxon signed-rank test (H₀: median ≤4). Statistical significance was set at *P*<.05 for all analyses.

### Ethical Considerations

This study did not involve human participants or patient-identifiable data. The Ethics Committee of Teikyo University reviewed the project and determined that formal ethical approval was not required because the work evaluated the quality of test items and did not constitute human medical research. Accordingly, informed consent was not applicable.

## Results

### Answer Accuracy

The accuracy of the LLMs on the full 200-item set and the nonimage 173-item set is shown in Table 3. All models consistently scored lower in the full set versus the nonimage set, with OpenAI o3 achieving the best results at 90% and 92.5%, respectively. A significant difference was seen between OpenAI o3 and OpenAI o4-mini on the full set, whereas no significant differences were seen among models on the nonimage set.

**Table 3.** Model accuracies and statistical comparisons on 200 benchmark questions and 173 nonimage questions.

Variables	200 questions^a^	173 nonimage questions^a^
Model accuracy		
OpenAI-o4-mini-high, %	86.0 (80.5, 90.1)	88.4 (82.8, 92.4)
OpenAI-o4-mini, %	82.5 (76.6, 87.1)	86.7 (80.8, 91.0)
OpenAI-o3, %	90.0 (85.1, 93.4)	92.5 (87.6, 95.6)
Gemini 2.5 Flash, %	83.0 (77.2, 87.6)	89.6 (84.1, 93.3)
Cochran Q test (*P* value)	.01	.10
Pairwise McNemar test (Bonferroni-adjusted *P* value)		
OpenAI-o4-mini-high versus OpenAI-o4-mini	≥.99	N/A^b^
OpenAI-o4-mini-high versus OpenAI-o3	.44	N/A
OpenAI-o4-mini-high versus Gemini 2.5 Flash	≥.99	N/A
OpenAI-o4-mini versus OpenAI-o3	.02	N/A
OpenAI-o4-mini versus Gemini 2.5 Flash	≥.99	N/A
OpenAI-o3 versus Gemini 2.5 Flash	.06	N/A

a Accuracy shown with 95% CIs in parentheses (Wilson score, two-sided, without continuity correction).

b Not applicable.

### Item Generation

**Figure 2.**
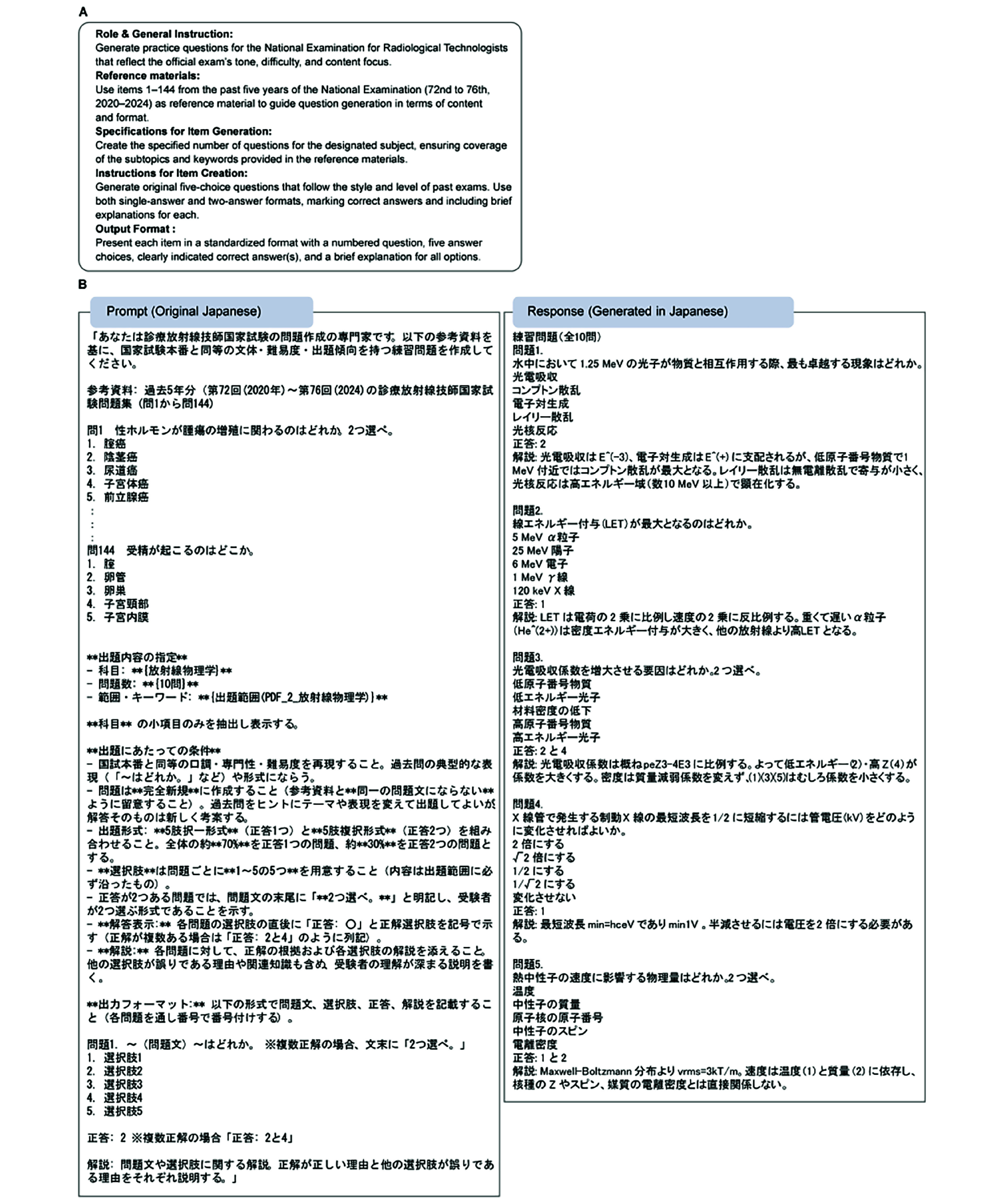
Prompt summary and representative example of item generation. (A) Summary of the prompts used to instruct the language model to generate original mock questions aligned with the National Examination for Radiological Technologists. The summary outlines the role of the model, input references, specifications of generation, item-creation rules, and output format. (B) The actual prompt and representative response generated by the model. The prompt included specific formatting and content-generation instructions written in Japanese. The response shows the generated item, correct answers, and explanation in Japanese.

Table 4 presents the scores and statistics for all 192 questions, while Figure 2 illustrates the prompt template and sample outputs. Among item difficulty, factual accuracy, and accuracy of content coverage, the medians and the proportions of scores ≥4 did not differ significantly, although accuracy of content coverage had the highest score. Meanwhile, instructional usefulness had a significantly lower score than appropriateness of wording. The evaluation criteria and evaluation examples of items that scored 1‐2 for the lower-scoring criteria—appropriateness of wording and instructional usefulness—are detailed in Multimedia Appendix 2.

**Table 4.** Reviewer ratings by evaluation criterion for the AI-generated items (n=192).

Evaluation criterion^a^	Mean score (95% CI)	*P* value^b^
Item difficulty	4.29 (4.11, 4.46)	<.001
Factual accuracy	4.18 (3.98, 4.38)	.001
Accuracy of content coverage	4.73 (4.60, 4.86)	<.001
Appropriateness of wording	3.92 (3.73, 4.11)	.44
Instructional usefulness	3.60 (3.41, 3.80)	≥.99

a “Evaluation criterion” refers to the five evaluation criteria defined in Table 2.

b One-sided Wilcoxon signed-rank test against the null hypothesis such that the median score is ≤4.

## Discussion

### Principal Findings

This study compared four LLMs in terms of answer accuracy on the Japanese National Examination for Radiological Technologists. The top performer, OpenAI o3, was used to generate the mock test, which was then evaluated by experts in terms of educational quality. As shown in Table 3, on the full set of items, only the comparison between the OpenAI o3 and OpenAI-o4-mini variant reached statistical significance; when image-based items were excluded, no model differences were observed.

To contextualize the observed accuracy differences, we briefly summarize the multimodal architectures and vision–language pipelines of the evaluated models as they pertain to radiologic image questions. Built on a GPT-4 lineage, OpenAI o3 integrates a high-resolution visual encoder with unified attention over linguistic and visual tokens [18,19], likely enhancing sensitivity to low-contrast findings and subtle anatomical cues typical of radiography and CT. In contrast, OpenAI o4-mini is a lightweight variant with reduced-resolution patch embeddings [20,21], which can yield coarser visual representations and miss subtle image cues. OpenAI o4-mini-high supplements the mini architecture with targeted medical-image fine-tuning and partial recovery of high-resolution inputs [22,23], consistent with improved mapping of relevant visual patterns. Lastly, Gemini 2.5 Flash uses a two-tower design in which an external vision encoder converts images to tags prior to language processing [24,25]; such pipelines may incur information loss for domain-specific anatomical details. In line with these architectural differences, performance gaps emerged on image-based questions but not on text-only items.

The pronounced performance spread on image-based questions could be mainly attributed to the aggressive parameter reduction in OpenAI o4-mini and the information loss inherent in the image-to-tag pipeline in Gemini, both of which weaken visual feature representation. Thus, current systems may not fully capture clinically grounded context and the knowledge required for radiologic image interpretation. This finding is consistent with the results of previous studies reporting similar limitations in specialty radiology examinations [26,27]. However, OpenAI o3 and o4-mini-high have higher resolution encoders and benefit from medical-specific fine-tuning. However, due to the limited sample sizes and proprietary nature of the detailed model architectures, these explanations remain partly hypothetical. Nevertheless, these findings highlight the importance of the visual module scale and the presence of medical-domain training when selecting an LLM for the development of -generated questions in this field.

Building on these findings, the 192 items generated by the top model were reviewed across five educational criteria. Item difficulty, factual accuracy, and content coverage were rated favorably, indicating alignment with national expectations and the official blueprint [26]. By contrast, appropriateness of wording and instructional usefulness were comparatively weaker, with reviewers noting ambiguous phrasing and explanations that did not consistently link stem cues to the correct answer or to distractor misconceptions. These strengths and weaknesses are consistent with observations from related medical-education settings [28-30] and underscore the need for editorial refinement prior to instructional deployment.

This study has several limitations. First, the image-based items were excluded from expert review, thus precluding the assessment of visual tasks. Second, each question was evaluated by a single expert, and thus inter-rater reliability could not be assessed. Third, reproducibility is limited by the use of publicly accessible browser interfaces. All evaluations were conducted through browser UIs with visible labels: OpenAI o3, OpenAI o4-mini, OpenAI o4-mini-high, and Gemini 2.5 Flash. Although this choice mirrors typical educational use and simplifies image I/O, it limits control over versioning and decoding parameters. Prompt delivery also varied across platforms due to UI constraints: OpenAI models received items in 50-question batches per session, whereas Gemini required per-item input, with a single image upload when applicable. Such differences in prompt granularity, context priming, and file-attachment workflows may have influenced outputs and should be considered when interpreting the comparable performance of Gemini Flash and o3. To mitigate these effects, we used an identical Japanese prompt template, disabled memory features, initiated new sessions for each batch, preserved the original exam order, and performed a single pass per item without retries. Input handling is detailed in the Methods section. These input structures reflected platform UI constraints (OpenAI allowed 50-question batches per session, whereas Gemini required per-item prompts and a single image attachment when applicable); although memory features were disabled and each batch began in a new session, processing the OpenAI items in batches could still introduce minor within-session priming; therefore, residual order effects cannot be fully excluded. Application-level temperature settings were not user-configurable. Moreover, because decoding remained stochastic and we performed a single pass per item without retries, run-to-run response variability cannot be fully excluded even with identical prompts. Given that browser-based services can update without notice, outputs may drift over time even when identical prompts and labels are used [31-33]. Thus, to strengthen version control and reproducibility, future studies should standardize prompt injection through Application Programming Interface endpoints with pinned model snapshots, identical per-item wrappers, and fully logged metadata (prompt templates, model identifiers, timestamps, and decoding parameters). In the future, visual encoders are expected to operate at a higher resolution and undergo additional tuning for medical domains. This could enable LLMs to automatically generate image-based items across modalities (eg, computed tomography, magnetic resonance imaging, and ultrasound), thus bringing mock exams closer to clinical reality. Further improvements in the feedback system could also be seen. By delivering adaptive feedback that varies in depth according to each learner’s proficiency, students can be provided with on-demand, targeted remediation material. LLMs could also be used to map items to the national blueprint in real time, enabling the detection and correction of domain imbalances while reducing faculty workload. Lastly, aligning these models with overseas licensure frameworks could expand their use to ultimately support a multilingual, multi-profession, international mock-exam bank.

### Conclusions

This study demonstrated that an LLM (OpenAI o3) can attain high accuracy on national radiological technology examination, as well as generate new multiple-choice items with appropriate difficulty, factual correctness, and syllabus coverage, as evaluated by experts. Although the AI-generated questions fell short in terms of wording clarity and pedagogical feedback, these can be mitigated through targeted editorial review. Practically speaking, LLMs can be used to draft content that is eventually refined by the faculty. This workflow could enable the more efficient development of mock examinations and reinforce curriculum alignment without imposing additional burden on instructors. However, performance gaps on image-based items, the absence of inter-rater reliability data, and the inherent volatility of cloud-hosted models underscore the need for cautious implementation and transparent reporting of model metadata. Nevertheless, future advancements in high-resolution visual encoders and medical-specific tuning can close this multimodal gap, while adaptive feedback functions and automated blueprint mapping can further extend the educational value of AI-generated assessments. After overcoming these barriers in terms of technical improvements and reproducibility safeguards, LLMs can be a strong asset in radiological technology education, which can even extend to the licensure preparations of other allied health professionals worldwide.

## Supplementary material

10.2196/81807 Multimedia Appendix 1 Breakdown of the 2025 Japanese National Exam Questions by Subject.

10.2196/81807 Multimedia Appendix 2 Operational definitions and decision rules for item evaluation.

## Abbreviations

AI: artificial intelligence
LLM: large language model
UI: user interface
