# Supplementary material for: Large Language Models for the National Radiological Technologist Licensure Examination in Japan: Cross-Sectional Comparative Benchmarking and Evaluation of Model-Generated Items Study
Source: JMIR Med Educ. 2025 Nov 13;11:e81807. doi: 10.2196/81807 (PMC12614397; doi:10.2196/81807)
Supplement: Multimedia Appendix 1 [file mededu-v11-e81807-s001.docx]

**Multimedia Appendix S1. Breakdown of the 2025 Japanese National Exam Questions by Subject**

| **Subject** | **Questions (n)** | **Composition (%)** | **Items w/ Images (n)** |
| --- | --- | --- | --- |
| Diagnostic Imaging Techniques | 20 | 10 | 12 |
| Nuclear Medicine Technology | 20 | 10 | 1 |
| Radiation Therapy Technology | 20 | 10 | 1 |
| Medical Imaging Informatics | 10 | 5 | 1 |
| Healthcare Safety Management | 8 | 4 | 0 |
| Basic Medical Sciences | 30 | 15 | 0 |
| Radiation Science & Engineering | 36 | 18 | 2 |
| X-ray Imaging Equipment | 20 | 10 | 0 |
| X-ray Imaging Techniques | 20 | 10 | 9 |
| Image Engineering | 6 | 3 | 1 |
| Radiation Safety Management | 10 | 5 | 0 |
| Total | 200 | 100 | 27 |
