# Supplementary material for: Large Language Models for the National Radiological Technologist Licensure Examination in Japan: Cross-Sectional Comparative Benchmarking and Evaluation of Model-Generated Items Study
Source: JMIR Med Educ. 2025 Nov 13;11:e81807. doi: 10.2196/81807 (PMC12614397; doi:10.2196/81807)
Supplement: Multimedia Appendix 2 [file mededu-v11-e81807-s002.docx]

**Multimedia Appendix S2. Operational definitions and decision rules for item evaluation**

| Evaluation criterion | 5 (Adoptable) | 4 (Minor revision) | 3 (Revisable) | 2–1 (Major revision/unacceptable) |
| --- | --- | --- | --- | --- |
| Item difficulty | Difficulty clearly appropriate; plausible distractors; no cueing. | Slightly easier/harder but acceptable. | Noticeably too easy/hard (e.g., trivial recall or niche subspecialty detail) yet fixable by editing a cue, stem specificity, or distractor set. | Fundamentally mistargeted or not correctable without rewriting. |
| Factual accuracy | All statements are accurate and current. | Minor wording/unit-precision issues (e.g., “~” vs exact threshold) that do not change the key. | A distractor contains a partially true statement likely to mislead; fixable by rephrasing. | The answer key is incorrect, or multiple options could be correct. |
| Accuracy of content coverage | Directly maps to a listed objective; correct cognitive level. | Slight drift in scope but still within the same subdomain. | Crosses into another subject or mixes objectives; fixable by retargeting the stem. | Off-blueprint or duplicated content. |
| Appropriateness of wording | Clear, concise, idiomatic; no ambiguity. | Minor edits improve flow (e.g., replace “often/commonly” with quantifiable terms); generic and vendor terms are both present and defined. | Ambiguity or double negative that could change interpretation; fixable by rewording, including cases where vendor-specific or undefined technical terms are used without the generic concept. | Confusing or misleading language; requires rewrite. |
|  | [Example \| Score 2: Appropriateness of wording]  Question: Which modification to fMRI acquisition is effective for reducing geometric distortion in BOLD fMRI while maintaining temporal resolution? Select two.  1. Multiband (MB) EPI  2. RESOLVE (readout-segmented EPI)  3. 3D-TSE  4. VIBE (volumetric interpolated breath-hold examination)  5. Balanced steady-state free precession  Generated explanation: Multiband echo-planar imaging shortens TR via simultaneous multi-slice excitation and helps suppress distortion. RESOLVE uses segmented EPI to split the readout time, which reduces chemical shift and geometric distortion. 3D-TSE and VIBE are not intended for BOLD applications. Balanced steady-state free precession is sensitive to magnetic susceptibility.  Our note: The stem and options mix vendor-specific names (e.g., RESOLVE and VIBE) with generic terms without first presenting the generic concepts (e.g., readout-segmented EPI and 3D spoiled GRE). | | | |
| Instructional usefulness | Explains why the key is correct and why each distractor is plausible but wrong, with references to stem cues. | Clear rationale for the key; brief notes for most distractors. | Generic or fact-only rationale; limited linkage to the stem; fixable by adding cue-based reasoning. | Vague restatement or incorrect pedagogy. |
|  | [Example \| Score 1: Instructional usefulness]  Question: Which radiopharmaceuticals are likely to cause a “metallic taste” and a “sensation of warmth” immediately after injection? Select two.  1. ^99m^Tc-HMDP  2. ^123^I-IMP  3. ^99m^Tc-MIBI  4. ^131^I-NaI  5. ^18^F-FDG  Generated explanation: ^123^I-IMP and ^99m^Tc-MIBI are lipophilic and rapidly distribute to high-flow tissues, such as the brain and myocardium, which can transiently cause a metallic taste or a sensation of warmth in the mouth or throat. The reported frequency is lower for the other agents.  Our notes: The answer key designates choices 2 and 3 as correct; however, according to the PMDA database and the official package insert, 123I-IMP is not associated with a metallic taste or a sensation of warmth. | | | |
